# Sources of variation in false discovery rate estimation include sample size, correlation, and inherent differences between groups

**DOI:** 10.1186/1471-2105-13-S13-S1

**Published:** 2012-08-24

**Authors:** Jiexin Zhang, Kevin R Coombes

**Affiliations:** 1 Department of Bioinformatics and Computational Biology, The University of Texas MD Anderson Cancer Center, Houston, Texas 77030, USA

## Abstract

**Background:**

High-throughtput technologies enable the testing of tens of thousands of measurements simultaneously. Identification of genes that are differentially expressed or associated with clinical outcomes invokes the multiple testing problem. False Discovery Rate (FDR) control is a statistical method used to correct for multiple comparisons for independent or weakly dependent test statistics. Although FDR control is frequently applied to microarray data analysis, gene expression is usually correlated, which might lead to inaccurate estimates. In this paper, we evaluate the accuracy of FDR estimation.

**Methods:**

Using two real data sets, we resampled subgroups of patients and recalculated statistics of interest to illustrate the imprecision of FDR estimation. Next, we generated many simulated data sets with block correlation structures and realistic noise parameters, using the Ultimate Microarray Prediction, Inference, and Reality Engine (UMPIRE) R package. We estimated FDR using a beta-uniform mixture (BUM) model, and examined the variation in FDR estimation.

**Results:**

The three major sources of variation in FDR estimation are the sample size, correlations among genes, and the true proportion of differentially expressed genes (DEGs). The sample size and proportion of DEGs affect both magnitude and precision of FDR estimation, while the correlation structure mainly affects the variation of the estimated parameters.

**Conclusions:**

We have decomposed various factors that affect FDR estimation, and illustrated the direction and extent of the impact. We found that the proportion of DEGs has a significant impact on FDR; this factor might have been overlooked in previous studies and deserves more thought when controlling FDR.

## Introduction

With the advent of high throughput technologies, research has focused on the systematic genome-wide study of biological systems. Microarray technology has been used to measure the mRNA expression of thousands of genes simultaneously. Concurrently, new statistical methods have been developed to analyze the data generated by these experiments. These methods involve both data preprocessing (background correction, data transformation, normalization, etc.) and specific tools for different types of studies (e.g., class discovery, class prediction, or class comparison).

The canonical class comparison problem involves the identification of lists of DEGs. The evolving consensus [1] on the analysis of microarray data recognizes the centrality of methods that estimate the FDR associated with gene lists. Although the concept of FDR was introduced by Benjamini and Hochberg in 1995 [2], a variety of methods have been introduced since then to estimate the FDR in microrray data sets [3-8]. These methods share certain characteristics: they perform a separate statistical test for each gene or protein; they compute a *p*-value associated with each test; and they estimate the FDR using the distribution of *p*-values. The methods are usually based on the assumption of independent or weakly dependent test statistics. However, when dealing with microarray data, we know that genes are usually correlated either for biological or technical reasons. Recent studies have demonstrated the non-negligible effects of correlation in microarray data on large-scale simultaneous hypothesis testing and pointed out how variable the FDR could be in the presence of strong correlation [9-13].

Our study sheds more light on possible reasons for the (lack of) precision in the estimated FDR. Our results provide two concrete examples of this imprecision. First, we look at an example where univariate Cox proportional hazards (CPH) models are used to determine which genes appear to be related to survival. By resampling ~ 100 patients at a time (out of a set of ~ 200 patients), we find that the estimates of the percentage of genes that appear to be related to survival range from 0% to 20%. In a simpler example of univariate t-tests between two groups of samples, we find that the estimate of the percentage of DEGs ranges between 13% and 43%. This range of estimates is much wider than one would anticipate from fitting a distribution based on thousands of *p*-values. However, those *p*-values are not independent. Since gene expression is often correlated, the effective number of independent measurements used to estimate the distribution may be quite small. Strategies that have been proposed to improve the estimation of FDR include resampling [11,14,15] and latent FDR with a random term to capture the correlation [12]. However, the causes of highly variable FDR estimation are multifold and deserve further investigation.

Throughout this paper, we estimate FDR using a BUM model for the distribution of *p*-values [3]. We select this method primarily because it can be computed quickly. While the method may not be the most accurate way to estimate FDR, the fact that it gives a relatively good fit to the *p*-value distributions that we encounter suggests that our results are driven by intrinsic variability in the *p*-value distributions from one sample set to another, and thus are likely to affect all known methods to estimate FDR.

In many cases, it is difficult to evaluate analytical methods for microarray data because of the complex— and unknown—nature of the underlying biological phenomena. Thus, simulated data sets with known “ground truth” are needed in order to assess the performance of computational algorithms for the analysis of high throughput data. To address this problem, many groups have developed microarray simulation software [10,16-20]. However, many existing simulations rely on simplified ideas of the underlying biology. For instance, the manuscript [10] that introduced the SPLOSH method to estimate FDR includes a simulation that assumes that (i) genes are independent and (ii) the genes that are differentially expressed all have the same fold change. Neither assumption is likely to hold in the real world, and these simplified assumptions do not give a realistic view of the variability in the FDR estimates.

We have already introduced a package of microarray simulation software called UMPIRE [21]. The current version of UMPIRE allows researchers to simulate heterogeneous microarray data with correlated block structure, which is linked to binary or time-to-event outcomes. Through a comprehensive set of simulations, we show that sample size, correlation structure and portion of DEGs account for the majority of observed variability in the *p*-value distributions and FDR estimates found in real data.

## Methods

### Public data sets

The Affymetrix microrray data were collected as part of a study to predict survival in follicular lymphoma patients [22]. The data set contains 191 patient samples measured with Affymetrix U133A and U133B arrays. Dave and colleagues quantified the microarray data using Affymetrix MAS 5.0 software and then transformed the results by computing the base-two logarithm. They also separated the samples into training (*N* = 95) and testing (*N* = 96) sets. We downloaded the processed microarray data and associated clinical annotations from the supporting web site (http://llmpp.nih.gov/FL/).

The two-color fluorescent cDNA microarray data were collected as part of a study to identify clinically relevant subtypes of prostate cancer [23]. The data set consists of data from 41 healthy prostate specimens, 62 primary prostate tumors, and 9 unmatched lymph node metastases. The microarrays contain 42,129 spots for 38,804 different cDNA clones representing 21,287 distinct UniGene clusters. The prostate cancer samples were labeled with Cy5. A common reference material, pooled from 11 established human cell lines, was labeled with Cy3. The raw microarray data were downloaded from the Stanford Microarray Database (http://cmgm.stanford.edu/pbrown/). We used intensity-dependent loess normalization to normalize the background-corrected channels from each microarray, after which the log ratios between experimental and reference channels were transformed by computing the base-two logarithm.

### Simulated data sets

Genes could be correlated when they are involved in active biological pathways, or are regulated by the same set of factors. We consider the correlation in gene expression to be “clumpy”, meaning that there are gene groups with high correlation within groups but no or little correlation between groups. In order to mimic this correlation feature, we applied block structure. Both the block sizes and the correlations within a block vary in order to mimic different sized pathways/networks, and loosely or strongly correlated genes within a particular pathway/network. Distinct blocks are assumed to be independent. Please refer to our previous publication for detailed description of the block structure [21].

Using the UMPIRE package, additive and multiplicative noise were incorporated, and correlated blocks were implemented. We simulated normal samples as a homogeneous population with *G* genes and *N* samples. We allowed *N* to vary in order to study the effect of the number of independent observations on various test statistics. The same number of cancer samples were generated with a portion of DEGs, where differential expressions were simulated as changed mean expression. Rather than focusing on individual genes, we altered the means of blocks of genes in order to mimic the effect of cancer pathology on pathways or networks. We let *ѱ* denote the percentage of differentially expressed blocks, which were randomly selected from the transcriptionally active blocks. Although it is possible for an inactive block of genes in normal samples to be turned on in cancer samples, or vice versa, we kept transcriptionally inactive blocks inactive in both normal and cancer samples in this simulation. The absolute changes of the mean expression values on log scale for a block of genes were given by Δ*_g_* ~ Gamma(*α*, *β*)*.* Both parameters for this gamma distribution were set to 10 so that the absolute fold change on the log2 scale was 1, and the long tail on the right hand side of the distribution allowed a few genes to have large fold changes. A gene in the changed block was randomly assigned to be up-regulated or down-regulated in cancer samples.

## Results

### Survival in follicular lymphoma

**Figure 1:**
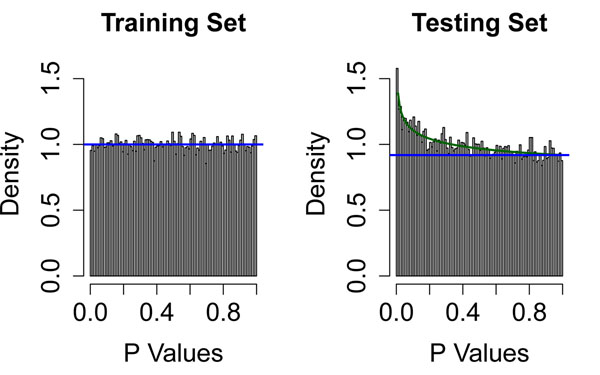
**Distribution of gene-by-gene *p*-values from follicular lymphoma data.** Histogram of gene-by-gene *p-*values from univariate Cox proportional hazards models in the training (left) and testing (right) sets of follicular lymphoma patients.

Dave and colleagues [22] collected Affymetrix U133A and U133B microarray data on samples from 191 follicular lymphoma patients for whom they also had extensive clinical follow-up. They split the data set into training (*N* = 95) and testing (*N* = 96) using a method that guaranteed that the survival curves for the two halves of the data would be the same. Their method for developing a model to predict survival began by computing *p-*values for each gene, derived from univariate CPH models in the training set. In order to better understand their method, we began our own analysis by fitting CPH models on both the training and testing sets. Figure 1 contains histograms of the resulting *p-*values. The distribution of *p-*values in the training set is uniform, which suggests that there is no evidence that any individual gene is associated with survival. However, the histogram of *p-*values in the test set shows clear evidence of an enrichment of small *p-*values, and is well-fit by a BUM model [3] that suggests that as many as 8% of the genes in the study are associated with survival. A similar histogram (data not shown) of the *p-*values from two-sample t-tests contrasting gene expression between training and testing sets was also uniform, suggesting that no genes were differentially expressed. We found this collection of results surprising: although the survival curves and the expression of individual genes were the same in training and testing sets, there appeared to be a difference in the ability of individual genes to predict survival. To resolve this conundrum, we hypothesized that the two distributions of *p-*values must actually be more variable than the histograms suggest. We repeatedly split the data into half, computed gene-by-gene CPH models on each half, and fit BUM models to the *p-*values. We found that the estimate of the number of significant genes range between 0% and 20%, with a peak near 10%.

### Two-sample t-tests comparing prostate tumor with normal prostate

**Figure 2:**
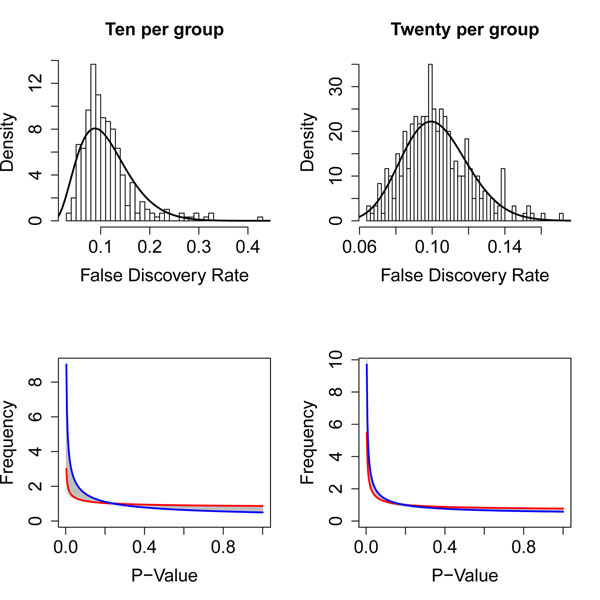
**FDR and BUM results from prostate data. (Top)** Distribution of the effective false discovery rate (FDR) at the median *p-*value cutoff when using ten (left) or twenty (right) samples per group in the prostate tumor vs. normal data set. **(Bottom)** Range of beta-uniform mixture (BUM) models of the distribution of *p-*values by randomly sampling ten (left) or twenty (right) samples per group. The red (resp., blue) curve marks the random samples with the smallest (resp., largest) estimated number of DEGs.

In order to study the variability of *p-*value distributions across experiments, we turned to a simpler example. Lapointe and colleagues [23] used two-color cDNA microarrays to study subsets of prostate cancer. Their study included 41 samples of normal prostate, 62 samples of prostate tumor, and 9 samples of lymph node metastases of prostate cancer. After downloading and processing the microarry data, we removed the lymph node metastases. We then repeatedly (300 times) subsampled the data set, randomly selecting ten normal and ten cancer samples each time. (We later repeated this analysis while selecting 20 samples per group.) Each time, we performed gene-by-gene two-sample t-tests to identify genes that were differentially expressed between normal prostate and prostate cancer, computed *p-*values, and fit a BUM model to the distribution of *p-*values. We found that the appropriate *p-*value cutoff to achieve an FDR of 10% varied between 1.6 × 10^–7^ and 0.0434 when using ten samples per group, with a median of 0.00628. The cutoff ranged from 0.0089 to 0.0439, with a median of 0.0229, when using twenty samples per group. If we tried to use the median cutoff across all random resamplings from the full data set, we also found that the effective FDR varied widely from one data set to another (Figure 2). This variability could be directly attibuted to differences in the distribution of *p-*values.

### Simulations

The two practical data sets were used to demonstrate the variability in the distribution of p-values and FDR estimation observed in real data. However, due to the limited sample size and unknown ground truth, the practical data sets lack the flexibility needed for testing analytical methods. Thus, realistic simulations were used to disentangle different factors contributing to the variation in FDR estimation.

We simulated 128 sets of normal and cancer data, using four different sample sizes (*N* = 10, 25, 50 and 100), eight different mean block sizes (*ξ* = 1, 5, 10, 50, 100, 250, 500, 1000), and four different *θ* scenarios, where *θ* denote the portion of negatively correlated genes within a block. In addition, we tested five levels of differential expression (*ѱ* = 0, 5%, 10%, 20%, and 40%) with three different mean block sizes (*ξ* = 1, 10, 100) and 25 samples in each biological condition. Our first goal was to study how sample size, block size, *ѱ*, and *θ* affect FDR. Based on the results, different *θ*s do not have a pronounced impact on various parameters of interest (data not shown). Thus in the following sections, we will only show the results obtained from *θ* that is uniformly distributed between 0 and 0.5 (*θ* = 0.5 – |*x –* 0.5| where *x* ~ Beta(1, 1)). We performed gene-by-gene two-sample t-tests in order to identify DEGs between normal and cancer samples. Then we modeled the *p-*values using a BUM model. Several parameters of interest were recorded for each data set.

#### Precision of parameters estimates in the BUM model

Pounds and Morris [3] showed that the distribution of *p-*values can be approximated by a BUM distribution, whose probability density function is:

for 0 <*x* ≤ 1, 0 <*α* < 1, and 0 < λ < 1. The parameter λ determines the size of the uniform component of the model. The shape of the distribution is determined by *α*; smaller values of *α* yield sharper peaks near zero. We estimated  and  for each simulation.

**Figure 3:**
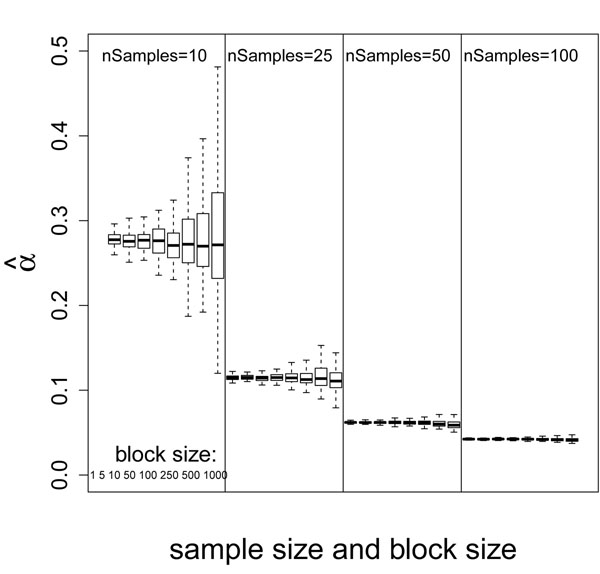
Boxplot of  grouped by sample size and block size

Both the mean and the variance of  decrease with larger *ѱ* (data not shown), but the effect is not dramatic. Figure 3 shows the distribution of  for different combinations of sample size and block size when *ѱ* = 10%. For a fixed sample size, the variance of  increases with larger block size, while the mean of  is unchanged. Because the genes within a block are correlated, larger block size results in fewer independent gene measurements. The estimation of parameters using fewer independent measurements should be less accurate and more variable. Thus, the phenomena of more variable  can be explained by the reduced number of independent measurements in data set with large block size. The constant mean of  indicates that the block size does not affect the average shape of the BUM model. On the other hand, for the same block size, both the mean and the variance of  decrease with larger sample size. The decreased variance of  is due to the increased estimation power with more observations (samples). The decreased mean of  indicates that the shape of the BUM model is getting steeper, which suggests the model predicts DEGs with more confidence. This is consistent with the increased power expected from more observations.

**Figure 4:**
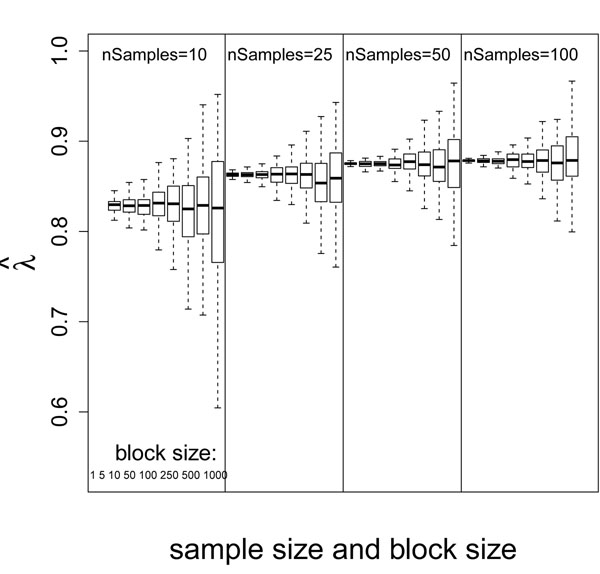
Boxplot of  grouped by sample size and block size

Figure 4 shows the distribution of  for different combination of sample size and block size when *ѱ* = 10%. Like , for the same sample size, the mean of  is essentially constant for different block sizes, and the variance of  increases for larger block size because we have fewer independent measurements. For the same block size, the variance of  also decreases for larger sample size because of more observations. Unlike , the mean of  increases with larger sample size. Note that we set *ѱ* (proportion of DEGs) equal to 0.1 in the simulations summarized in Figure 4. With the increased power from more observations, the uniform portion of the BUM model approaches the true proportion, 1 – *ѱ*, of non-DEGs. Since  represents the contribution of the uniform portion to the BUM model, larger *ѱ* results in smaller .

From  and , we can estimate the logical upper bound of *π* using the formula:

The remaining  portion of the set of *p-*values arises from the alternative hypothesis; that is,  represents the proportion of DEGs. In our example, the true value of *π* = 1 – *ѱ* = 0.9 since *ѱ* = 10%. The distribution of the calculated upper bound of  when *ѱ* = 10% has a pattern similar to the one shown in Figure 4. Even though the upper bound of  is approaching its true value with large sample size, the model appears to consistently underestimate the proportion of non-DEGs and overestimate the proportion of DEGs. This finding is consistent with previous studies [11].

We also studied the correlation between  and , which are negatively correlated (Additional file 1). However, the extent of negtive correlation decreases with increasing sample size. The negative correlation is due to the fact that the total area under the BUM model sums to 1. The smaller  is, the smaller the alternative component (beta density) and the larger the null component (uniform density) which corresponds to larger . With larger sample size, variabilities of  and  are reduced due to more independent observations, which explains the decrease in absolute correlation.

#### False discovery proportion as a function of p-value

We simulated cancer samples with a proportion of DEGs having altered mean expression values. For given (nominal) *p*-value thresholds, we counted the number of significant DEGs that were called “positive”. Because we know which genes were truly differentially expressed in the simulation, we calculated the number of true positives (TP) and false positives (FP) for each *p*-value threshold. Next, we calculated the observed false discovery proportion: *FDP* = *FP/*(*FP* + *TP*)*.* (FDR is the marginal average of the FDP.)

**Figure 5:**
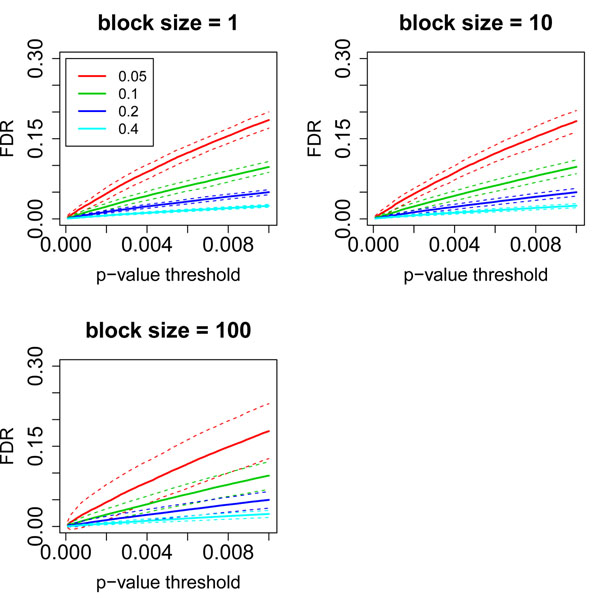
**FDP for different block sizes and *ѱ.*** Solid lines represent the mean FDPs for particular block size and *ѱ*, while dashed lines represent standard deviations of corresponding FDPs. Different colors are used to distinguish scenarios of different *ѱ*, as shown in the legend of the top-left figure.

One factor that dramatically affects FDP is *ѱ* (portion of DEGs). Figure 5 shows FDP estimated from different combinations of block sizes and *ѱ.* We observe clearly that larger *ѱ* is associated with smaller and better estimated FDP (less variable). This is not surprising because more true positives render more power during estimation. Another observation from Figure 5 is that block size only affects the variation of FDP estimation.

To illustrate the influence of sample size on FDP estimation, we performed another set of simulations with different sample sizes and block sizes, but fixed *ѱ* (10%). Since we know that the mean of FDP is not significantly affected by block size, we calculated mean FDP by averaging over all block sizes for each sample size. Additional file 2 shows that the mean FDP decreases with larger sample size. Consistent from what we observed in Figure 5, larger block size corresponds to more variable FDP estimation, presumably because of the decreased number of independent measurements.

#### Efron’s dispersion variate and the standard deviation of the correlation density

Efron [9] showed that correlations among genes could considerably widen or narrow the distribution of the test statistic under the null hypothesis. Moreover, the main effect of the pair-wise correlation could be summarized by a single dispersion variate *A.* The central peak of the null distribution widens when *A* > 0, and narrows when *A* < 0. We recorded *A* for each simulation; Additional file 3 shows the boxplots of *A* grouped by sample size and block size when *ѱ* = 10%. The mean value of *A* is essentially unaffected by either block size or sample size, but the variance of *A* increases with larger block size. This observation is consistent with Efron’s finding that more correlation leads to larger variance.

**Figure 6:**
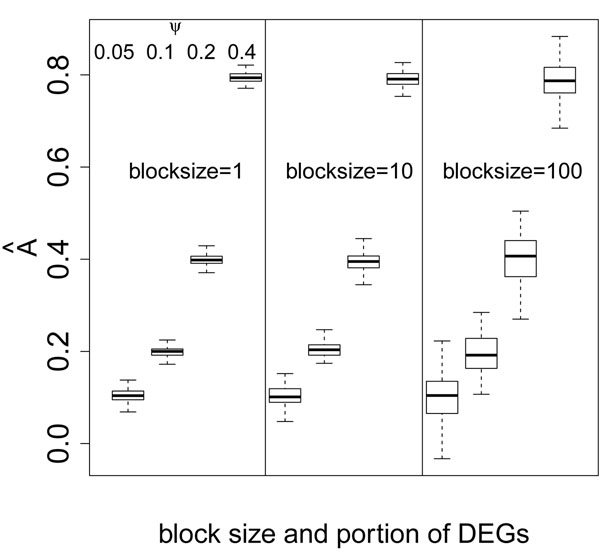
Boxplot of dispersion variate A under different combinations of block sizes and *ѱ*

One novel finding from our study is that the dispersion variate *A* is dramatically affected by *ѱ* (Figure 6). With larger *ѱ* (i.e., more DEGs), the distribution of the test statistic widens, so *A* is larger. The values of *Â* are almost always positive in this set of simulations, which is supposed to mean that gene correlations usually widen the distribution. However, Figure 6 clearly shows that the widening of the distribution is attributable to an increase in the proportion of true positives, and not to the increased amount of gene correlation.

**Figure 7:**
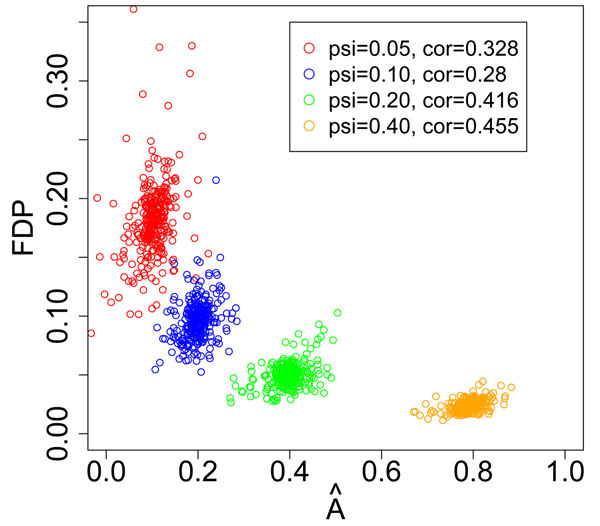
**Scatter plot of dispersion variate A and FDP.** Colors are used to distinguish results from different *ѱ.* The pearson correlations between dispersion variate A vs FDP under different *ѱ* are shown in legend.

We also found that FDP is negatively correlated with the dispersion variate *A*, which is the opposite of the conclusion in Efron’s paper. Figure 7 contains a scatter plot of *A* vs FDP for varying values of *ѱ.* For fixed *ѱ*, FDP and *A* exhibit weak positive correlation. However, when *ѱ* is allowed to vary, FDP and *A* are negatively correlated.

Also following Efron, we calculated the standard deviation of the empirical correlation densities (*corr.std*)*.* We found that *corr.std* increases with larger block size (Additional file 4). This result is not surprising, since increasing the block size increases the total amount of correlation present in the data but decreases the effective number of independent measurements that contribute to the estimate. However, the sample size has a much larger effect, with *corr.std* decreasing significantly with larger sample size.

## Conclusions

From the two concrete examples, we observed a lack of precision in the estimation of FDR. In order to study the sources of variation during FDR estimation, we simulated microarray data with more realistic parameters. In our simulation, block-wise structure with different block sizes and intra-block correlation are used to mimic the molecular networks or biological functional groups where large scale correlation of gene expression arises. A particular block of genes could be transcriptionally active or inactive depending on specific biological conditions. When the block of genes are transcriptionally active, their expression levels follow a multi-variate normal distribution with parameters estimated from real microarray data. Certain portion of genes will be differentially expressed between normal and cancer samples, where the magnitude of changes follows a gamma distribution which allows some large magnitude changes while the majority have two fold change on average. With this setting, we simulated microarray data sets with different sample sizes, correlation structure, portion of negtively correlated genes within a block, and portion of DEGs between two biological conditions.

For each simulated data set, we have recorded the parameters related to FDR estimation. Different portions of negtively correlated genes within a block do not affect the parameter estimations. Thus, the three major sources of variation in FDR estimation are the sample size, correlation structures and the portion of DEGs. With large sample size, the variances of all parameters decrease due to increased estimation power. However, the percentage of non-DEGs is always under-estimated, even though it approaches the true portion with larger sample size. Large block size results in less precise estimation of all the parameters due to less independent measurements. However, the block size does not affect the mean estimation of the parameters. Thus the FDR estimation are less precise with more correlation, but the average FDR estimation is not affected.

Our study suggests that an important factor affecting FDR estimation is the portion of DEGs. With larger portion of DEGs, the distribution of test statistic is widened by the larger portion of true positives, thus resulting in smaller FDR and more precise FDR estimation.

In summary, the correlation structure is not the only factor affecting FDR estimations. The portion of DEGs, which varies under different biological conditions contributes to both the precision and the magnitude of FDR estimation.

## List of abbreviations used

FDR: False Discovery Rate; FDP: False Discovery Proportion; UMPIRE: Ultimate Microarray Prediction, Inference, and Reality Engine; BUM: beta-uniform mixture; DEGs: differentially expressed genes; CPH: Cox proportional hazards

## Competing interests

The authors have no competing interests.

## Author’s contributions

JZ performed the simulations, gathered the simulation results, and completed the first draft of the manuscript. KRC initiated the project, provided ideas, developed UMPIRE, supervised the progression, and was involved in the manuscript development.

## Supplementary Material

Additional file 1Boxplot of pearson correlation between  and  for different sample sizesClick here for file

Additional file 2 **FDP for different sample sizes and block sizes** Solid black lines represent the mean FDPs from all simulated data for the same sample size. Dashed lines represent standard deviations of FDPs for different block sizes that are distinguished by colors shown in legend of bottom-right figure. Click here for file

Additional file 3 Boxplot of *Â* grouped by sample size and block size Click here for file

Additional file 4Boxplot of  grouped by sample size and block sizeClick here for file
