# Supplementary figures and images for: Sources of variation in false discovery rate estimation include sample size, correlation, and inherent differences between groups
Source: BMC Bioinformatics. 2012 Aug 24;13(Suppl 13):S1. doi: 10.1186/1471-2105-13-S13-S1 (PMC3426804; doi:10.1186/1471-2105-13-S13-S1)

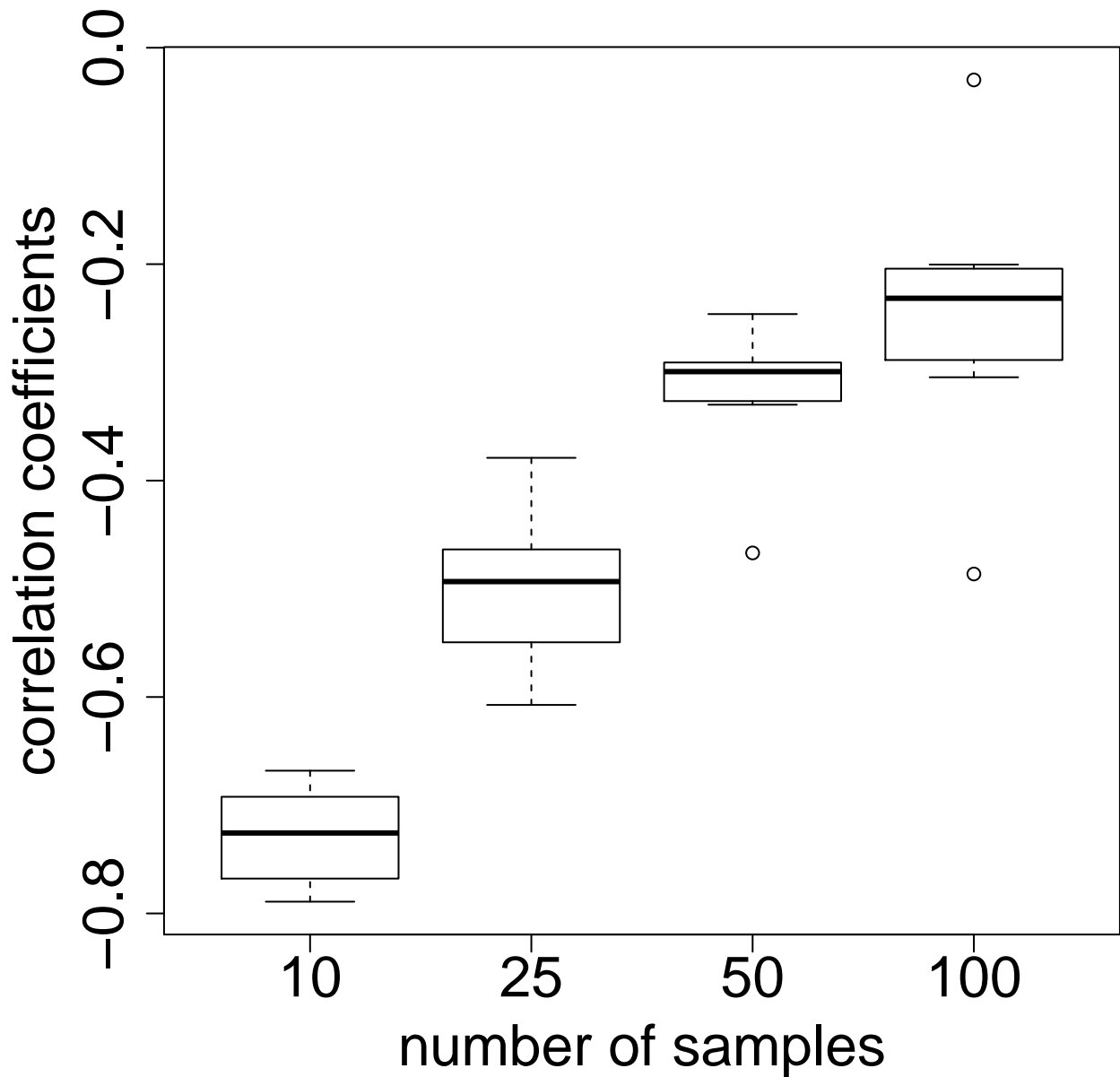

Supplement: Additional file 1 — Boxplot of pearson correlation between and for different sample sizes [file 1471-2105-13-S13-S1-S1.pdf]

**number of samples: 10**

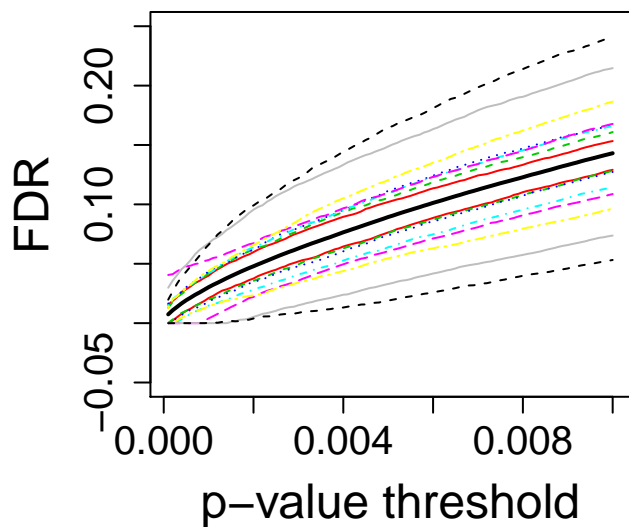

**number of samples: 25**

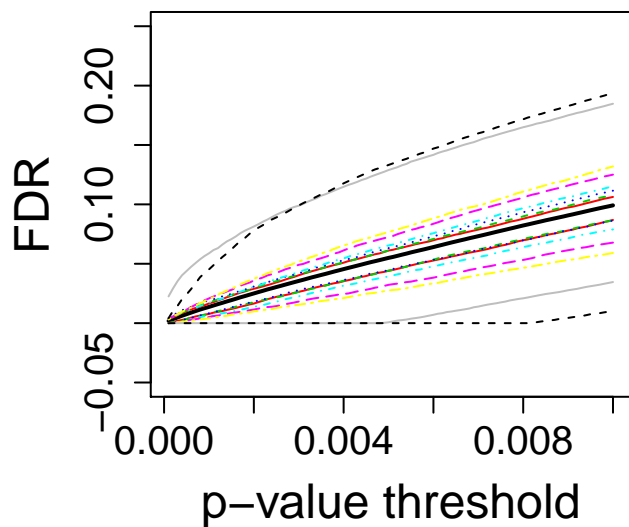

**number of samples: 50**

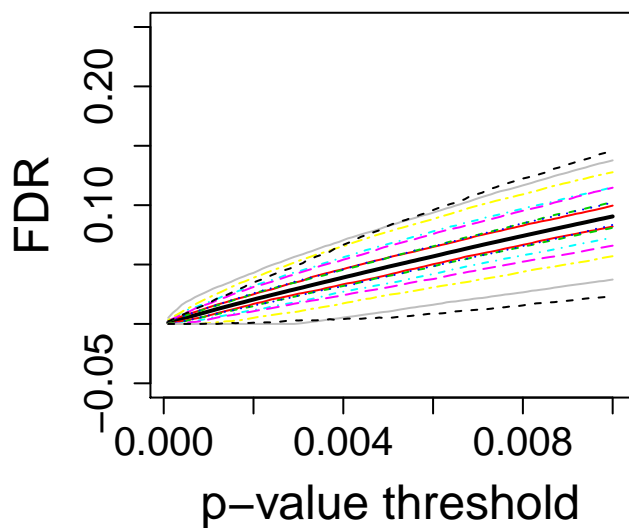

**number of samples: 100**

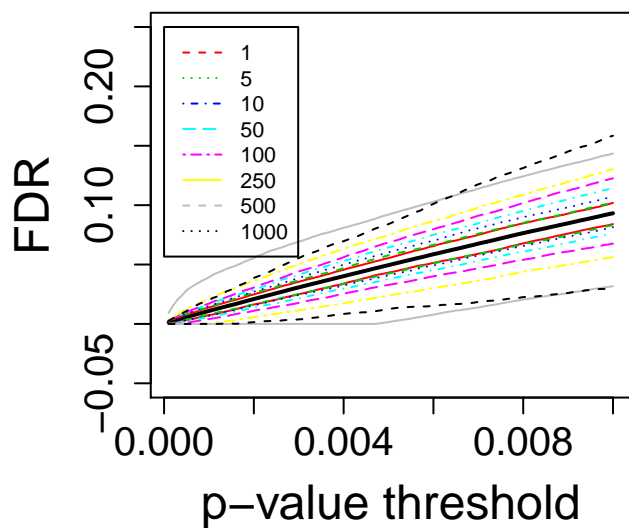

Supplement: Additional file 2 — FDP for different sample sizes and block sizes Solid black lines represent the mean FDPs from all simulated data for the same sample size. Dashed lines represent standard deviations of FDPs for different block sizes that are distinguished by colors shown in legend of bottom-right figure. [file 1471-2105-13-S13-S1-S2.pdf]

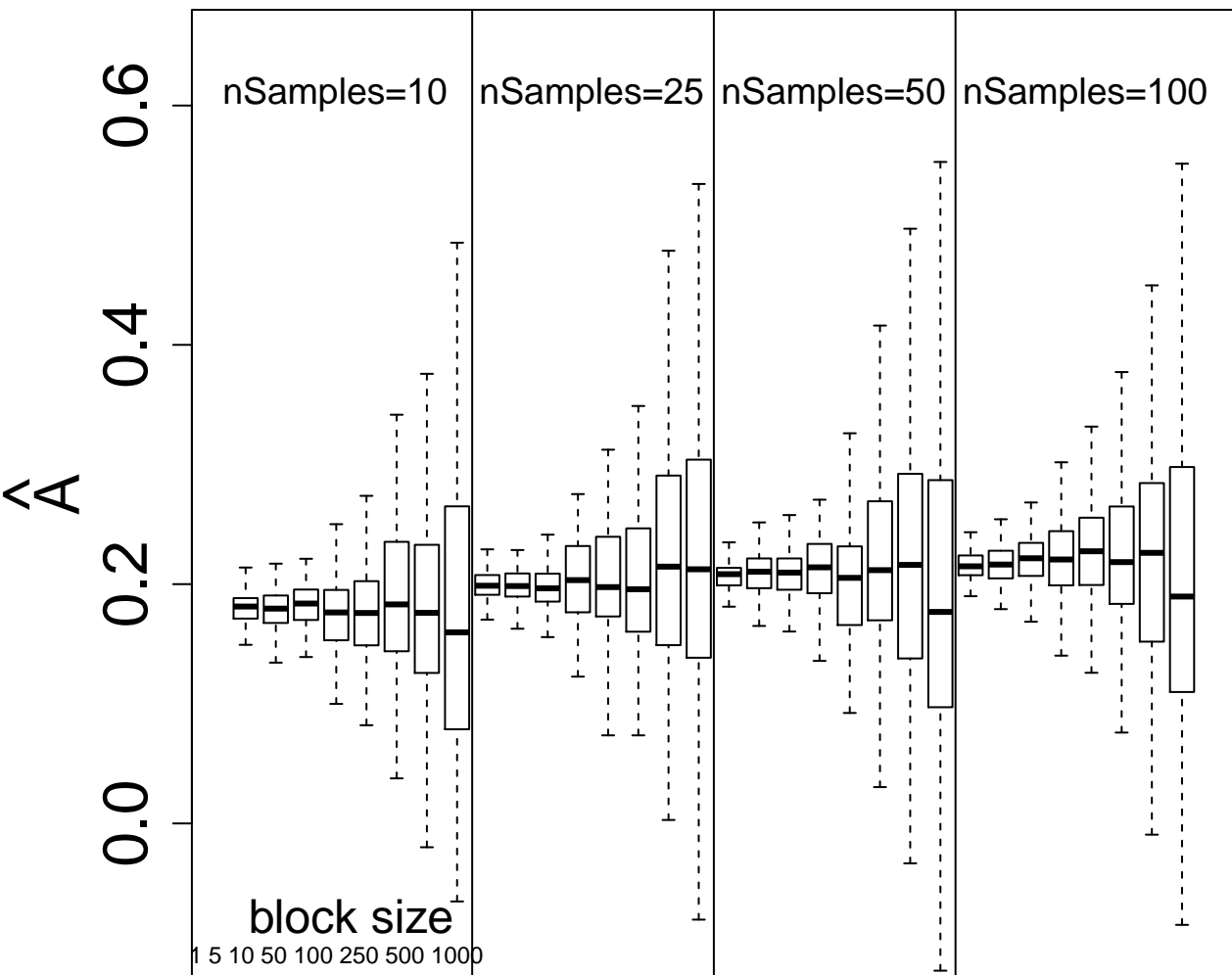

sample size and block size

Supplement: Additional file 3 — Boxplot of Â grouped by sample size and block size [file 1471-2105-13-S13-S1-S3.pdf]

corr.std

0.30

0.20

0.10

nSamples=10

nSamples=25

nSamples=50

nSamples=100

block size

1 5 10 50 100 250 500 1000

sample size and block size

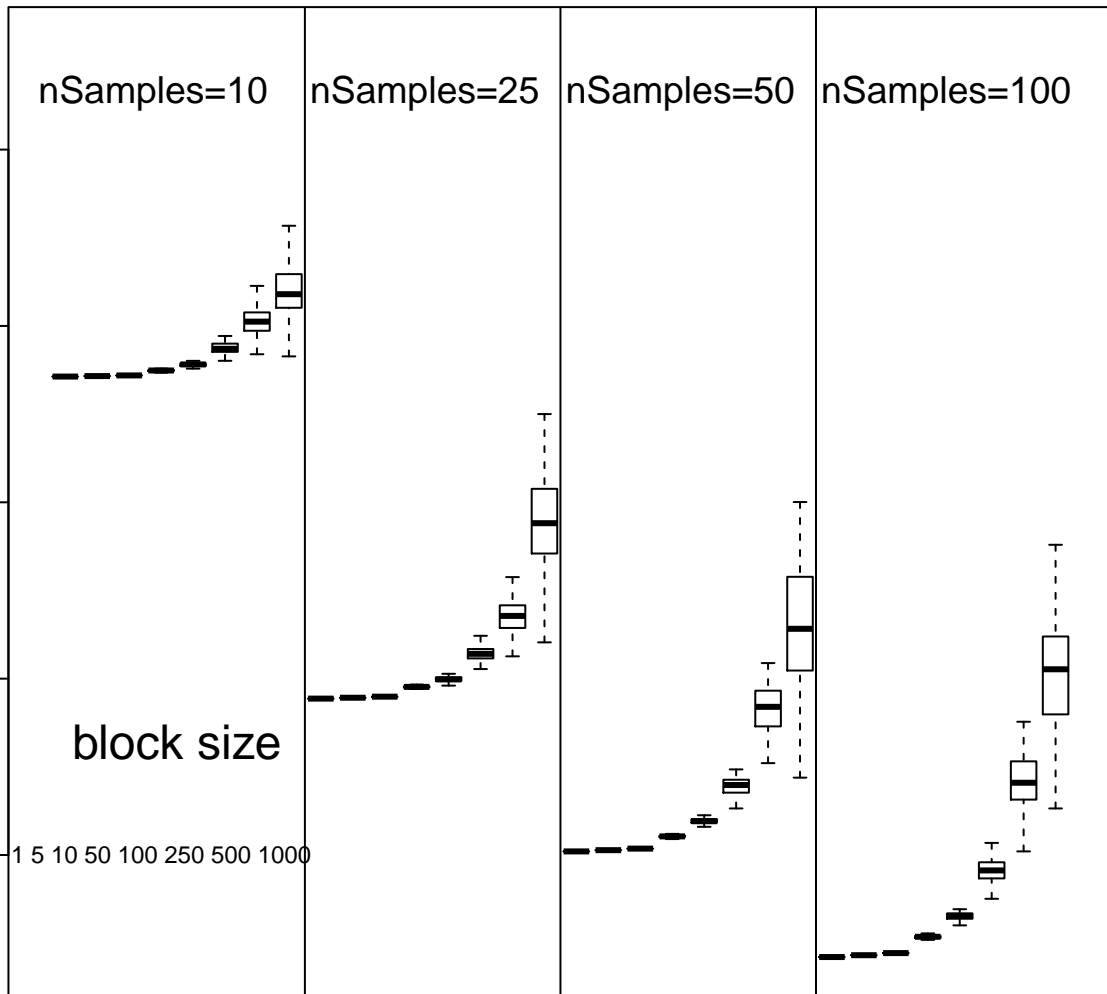

Supplement: Additional file 4 — Boxplot of grouped by sample size and block size [file 1471-2105-13-S13-S1-S4.pdf]
